# GWAS study using DNA pooling strategy identifies association of variant rs4910623 in *OR52B4* gene with anti-VEGF treatment response in age-related macular degeneration

**DOI:** 10.1038/srep37924

**Published:** 2016-11-28

**Authors:** Moeen Riaz, Laura Lorés-Motta, Andrea J. Richardson, Yi Lu, Grant Montgomery, Amer Omar, Robert K. Koenekoop, John Chen, Philipp Muether, Lebriz Altay, Tina Schick, Sascha Fauser, Dzenita Smailhodzic, Freekje van Asten, Eiko K. de Jong, Carel B. Hoyng, Kathryn P. Burdon, Stuart MacGregor, Robyn H. Guymer, Anneke I. den Hollander, Paul N. Baird

**Affiliations:** 1 Centre for Eye Research Australia, Royal Victorian Eye and Ear Hospital, Australia; 2 Ophthalmology, Department of Surgery, University of Melbourne, Australia; 3 Department of Ophthalmology, Donders Institute for Brain, Cognition and Behaviour, Radboud university medical center, Nijmegen, the Netherlands; 4 Statistical Genetics Laboratory, QIMR Berghofer Medical Research Institute, Brisbane, Australia; 5 Molecular Epidemiology, QIMR Berghofer Medical Research Institute, Brisbane, Australia; 6 Montreal Retina Institute, Westmount, Canada; 7 Paediatric Surgery, Human Genetics, and Ophthalmology, McGill University Health Centre, Montreal, Quebec, Canada; 8 Department of Ophthalmology, McGill University Health Centre, Montreal, Quebec, Canada; 9 Department of Ophthalmology, University Hospital of Cologne, Cologne, Germany; 10 Menzies Institute for Medical Research, University of Tasmania, Hobart, TAS and Dept: Ophthalmology, Flinders University, Adelaide, SA, Australia; 11 Department of Human Genetics, Radboud university medical center, Nijmegen, the Netherlands

## Abstract

Pooled DNA based GWAS to determine genetic association of SNPs with visual acuity (VA) outcome in anti-vascular endothelial growth factor (anti-VEGF) treated neovascular age-related macular degeneration (nAMD) patients. We performed pooled DNA based GWAS on 285 anti-VEGF treated nAMD patients using high density Illumina 4.3 M array. Primary outcome was change in VA in Early Treatment Diabetic Retinopathy Study (ETDRS) letters after 6 months of anti-VEGF treatment (patients who lost ≥5 ETDRS letters classified as non-responders and all remaining classified as responders). GWAS analysis identified 44 SNPs of interest: 37 with strong evidence of association (p < 9 × 10^−8^), 2 in drug resistance genes (p < 5 × 10^−6^) and 5 nonsynonymous changes (p < 1 × 10^−4^). In the validation phase, individual genotyping of 44 variants showed three SNPs (rs4910623 p = 5.6 × 10^−5^, rs323085 p = 6.5 × 10^−4^ and rs10198937 p = 1.30 × 10^−3^) remained associated with VA response at 6 months. SNP rs4910623 also associated with treatment response at 3 months (p = 1.5 × 10^−3^). Replication of these three SNPs in 376 patients revealed association of rs4910623 with poor VA response after 3 and 6 months of treatment (p = 2.4 × 10^−3^ and p = 3.5 × 10^−2^, respectively). Meta-analysis of both cohorts (673 samples) confirmed association of rs4910623 with poor VA response after 3 months (p = 1.2 × 10^−5^) and 6 months (p = 9.3 × 10^−6^) of treatment in nAMD patients.

Age-related macular degeneration (AMD) is a common complex progressive neurodegenerative disease in the elderly, which can lead to irreversible severe vision loss^1^. Vision is threatened when AMD advances to its late sequelae of either geographic atrophy (GA) or choroidal neovascularization (CNV) also known as nAMD^2^. One of the most important regulators of the neovascularization process is vascular endothelial growth factor A (VEGF-A)^3^. Currently, the most effective treatment for nAMD is inhibition of VEGF through the use of recombinant, humanised anti-VEGF monoclonal antibodies such as ranibizumab (Lucentis), aflibercept (Eylea) or off-label bevacizumab (Avastin). These drugs have been shown in multiple studies^4^,^5^,^6^,^7^,^8^ to be efficacious in improving vision, but a varying response to anti-VEGF treatment has been observed. Approximately 10% of patients showed no improvement in visual acuity (VA) (loss of >15 ETDRS letters), and exhibited a continuous decline in VA over two years of treatment similar to that previously reported for both ANCHOR and MARINA trials^5^,^6^. This range of variable VA response may in part be explained by genetic predisposition.

It is well established that genetic factors are associated with risk of developing AMD^9^. Several of the genes associated with AMD are also genes encoding components of the neovascularization pathway and have previously been investigated in variation to anti-VEGF treatment response studies^8^,^10^,^11^,^12^. However, conflicting findings have been reported thus far.

The current study aimed to investigate associations with genetic variants in a hypothesis-free manner using a genome-wide association study (GWAS). We investigated whether genetic factors influencing ranibizumab treatment outcomes in nAMD patients could be identified through the initial use of a GWAS pooling strategy followed by a technical validation and subsequent replication in an independent cohort.

## Results

The objective of this study was to investigate whether genetic variants could be identified that might influence the treatment outcome after anti-VEGF treatment in nAMD patients. The demographic characteristics of patients of the Melbourne discovery cohort and the replication cohort are shown in Table 1. A total of 297 patients from the Melbourne discovery cohort (285 individuals used initially in the pooled GWAS, plus an additional 12 ranibizumab-treated AMD patients were included, giving a total of 297 individuals) and 376 patients from the replication cohort met the study inclusion criteria with a mean age of 79.2 years and 77.1 years, respectively. A mean baseline VA (at the time of first injection) of 51 ETDRS letters was observed in the Melbourne discovery cohort and 52.6 ETDRS letters in the replication cohort (Table 1). In the Melbourne discovery cohort, sex, smoking, type of lesion, size of CNV and number of injections up to 6 months of treatment, showed no significant association with change in VA at 3 and 6 months of treatment (p > 0.05). Baseline VA showed a consistent negative association with change in VA at 3 and 6 months (p < 0.001) in both the Melbourne and replication cohort. In addition, age at the time of nAMD treatment was associated with change in VA at 3 months (p < 0.001) but not at 6 months in the replication cohort (p > 0.05) (Supplementary Table S1). Both the discovery and replication cohort have similar demographic and clinical characteristics except for the size of the CNV.

### First Phase- Genome wide association Study

**Figure 1:**
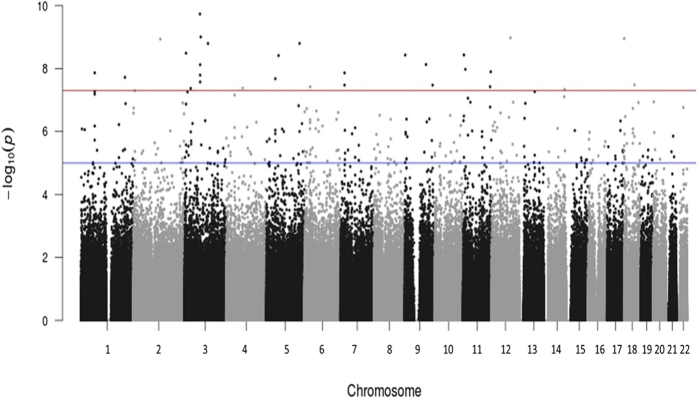
Manhattan plot of SNPs tested in a pooled GWAS for response to anti-VEGF treatment. The X-axis indicates the chromosomal position of the SNPs and the Y-axis shows their corresponding p-value (−log10). The red line indicates the threshold for genome-wide significance (p < 5 × 10^−8^) and the blue line indicates the threshold for suggestive association (p < 1 × 10^−5^).

First phase results from the pooled GWAS are shown in a Manhattan plot in Fig. 1. A total of 44 SNPs from the pooled GWAS were selected for technical validation through independent individual genotyping on the basis of a genome-wide significant p-value (37 SNPs, p < 9 × 10^−8^), SNPs in genes involved in drug resistance response (‘pharmagenes’) (2 SNPs, p < 5.0 × 10^−6^) and SNPs leading to a missense change in a coding region of a gene (5 SNPs, p < 1.0 × 10^−4^) (Table 2).

### Second Phase- Validation

In the second phase, all 44 SNPs from the first phase were genotyped in 297 patients (the same cohort as in phase 1 but with the addition of 12 extra patient samples who became available between the time of phase1 and phase2). This individual genotyping confirmed the 3 SNPs (rs4910623, rs323085, rs10158937) to be significantly associated (p < 0.05) with response at 6 months of ranibizumab treatment after Bonferroni correction for 44 independent tests. SNP rs4910623 showed the highest significance (p = 5.7 × 10^−5^), in which the ‘G’ risk allele led to a worse response (OR = 2.58 [95% CI = 1.63–4.10]). While the C allele of both rs323085 and rs10158937 (p = 6.5 × 10^−4^ and p = 1.30 × 10^−3^, respectively) appeared protective (OR = 0.16 [95% CI = 0.06–0.46] and OR = 0.32 [95% CI = 0.17–0.65], respectively) (Table 3).

Since a loading dose of three monthly injections is prescribed to all patients, we determined if the effect of these three selected SNPs in treatment outcome could be seen at this time point. The G allele of rs4910623 showed an association with worse outcome at three months of treatment (p = 1.5 × 10^−3^, OR = 2.23 [95% CI = 1.36–3.66]).

SNP rs10158937 also showed an association with the C allele leading to a better response after 3 months (p = 9.0 × 10^−3^, OR = 0.36 [95% CI = 0.17–0.77]), consistent with the six months result. However rs323085 did not show significant association at three months (Table 3).

### Third Phase- Replication

In the third phase, we analysed the association of the three SNPs with treatment outcome in a replication cohort of European descent, consisting of 215 ranibizumab-treated AMD patients following pro re nata treatment protocol. The effect of rs4910623 replicated at six months of treatment (p = 3.5 × 10^−2^, OR = 1.71 [95% CI = 1.04–2.80]). At the 3 month treatment time point all replication patients were included in the analysis (n  =  376) as they were all treated in a similar manner to the discovery cohort. The effect of this SNP on treatment response was also similar at this time point (p = 2.4 × 10^−3^, OR = 1.8 [95% CI = 1.24–2.71]). SNPs rs323085 and rs10158937 were not associated with treatment response in the replication cohort (Table 4).

**Figure 2:**
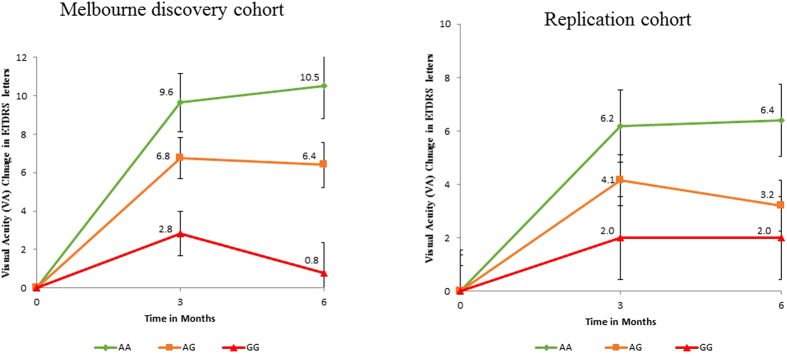
Mean change in visual acuity (VA) after three and also the six month time period (pro re nata treated only) of treatment stratified by rs4910623 genotype for the Melbourne discovery and replication cohorts. Vertical lines represent the standard error (SE) of the mean change in VA.

The frequency of rs4910623 risk allele (G) in the Melbourne discovery cohort was 71% and 72% for the non-responder group (3 and 6 months respectively) in comparison to 51% and 49% in the responders group (3 and 6 months, respectively) (Table 3). A similar change in allele frequency was also seen in the replication cohort with the frequency of the risk allele G being 62% and 38% in the non-responder group compared to 48% and 52% in the responders group (3 and 6 months (pro re nata patients), respectively) (Table 4). The overall distribution of change in VA corresponding to each genotype of rs4910623 in both cohorts is shown in Fig. 2.

### Meta-Analysis

A meta-analysis of SNP rs4910623 in both the Melbourne discovery cohort and the replication cohort showed differences in allele distribution between responders and non-responders at 3 months (p = 1.21 × 10^−5^) and 6 months (p = 9.3 × 10^−6^) of treatment (only pro re nata patients from the replication cohort were included) indicating the influence of rs4910623 on outcome of ranibizumab treatment in AMD patients (Table 5).

## Discussion

We sought to identify genetic variants that influence the ranibizumab treatment response in AMD. Using a pooled DNA GWAS approach, we identified SNP rs4910623 in the promoter region of the *OR52B4* gene as being associated with a worse response to ranibizumab treatment. The association was validated in the same cohort through individual genotyping and confirmed in an independent replication cohort of AMD patients of European descent. Individuals carrying the risk allele G did not respond as well to ranibizumab treatment in either cohort after 3 or 6 months of treatment compared to those with the A allele. In the replication cohort we observed significant association of rs4910623 with change in VA in patients on pro re nata treatment regimen at 6 months. However, 47 patients from Montreal were not included in this analysis as they followed a treat and extend regimen. We undertook an additional sensitivity analysis where these 47 patients were included back into the 6 month analysis time point to provide a total of 262 patients. A similar trend for association of the G allele of rs4910623 was seen (p = 4.6 × 10^−2^, OR = 1.53 [95% CI = 1.00–2.30]). As this group consisted of only 47 patients it was too small to draw any conclusive conclusions about whether this treatment protocol significantly affected the association of the G allele of rs4910623 with change in VA. In the meta-analysis we found significant association of rs4910623 patients with change in VA at both 3 and 6 months of treatment.

Two other SNPs, rs323085 and rs10158937, located *202* *kb 3*′ of *LOC100287225*and 42 kb at 3′ of *LEPR* gene respectively, did not remain associated with change in VA after replication. The inconsistency in the results for these two SNPs could indicate that they are false positive findings.

The baseline characteristics of both the Melbourne discovery and replication cohort did not reveal significant association with change in VA except for the baseline VA at 3 and 6 months of treatment. Baseline VA is negatively associated with change in VA, thus lower baseline VA will lead to a greater VA gain after treatment and vice versa. Therefore our findings are in agreement with the ANCHOR and MARINA trials where higher VA at baseline was associated with a smaller gain in VA at 12 and 24 months of treatment, respectively^13^. This in-turn reflects a floor and ceiling effect of baseline VA^14^. Based on definition of response in our study it may have floor effect of baseline VA where individuals with higher baseline VA will have limited chance to gain vision after the treatment and vice versa. On the other hand, the baseline VA is also reported as one of the predictors of treatment outcome for ranibizumab^15^. To overcome these potential issues we incorporated baseline VA as a covariate into our analysis. However, there was a difference in baseline CNV size in the discovery and replication cohort. This is probably due to differences in measurements between the cohorts with the Melbourne discovery cohort being based on one optic-disc area is equal to 2.54 mm^2^, using an optic disc diameter of 1.8 mm whereas the replication cohort was based on each patient’s optic disc area.

Using the HaploReg database^16^, SNP rs4910623 is predicted to alter the GATA and TCF4 regulatory motifs 22 bp upstream of the *OR52B4* gene. We also analysed the surrounding variants within its linkage disequilibrium (LD) block from the 1000 Genomes Project (r^2^ = 0.8, 250 Kb). A number of the identified SNPs in this LD block are predicted to alter transcriptional binding sites (Supplementary Table S3). Thus, this region may play an important role in transcriptional regulation of this gene.

The OR52B4 protein (Q8NGK2, UniprotKB accession number) consists of 314 amino acids (aa) and is a member of the G-coupled protein receptor (GPCR) family, which is located in the plasma membrane of the cell. Interestingly we find the conserved domain of Q8NGK2 17 is a 7-transmembrane domain (7tm) region which is highly conserved with the 7tm_4 super family domain of all the olfactory receptors (33–321 aa) and also the 7tm_1 domain of the 7 transmembrane receptor (rhodopsin family) (43–294 aa) as indicated using the NCBI Conserved Domain Database (CCD). (http://www.ncbi.nlm.nih.gov/Structure/cdd/wrpsb.cgi?seqinput=NP_001005161.2). Rhodopsin is also member of GPCR family and it functions to capture photons of light and trigger the intra-cellular photo-transduction cascade, forming the molecular basis of vision^18^. GPCRs are highly selective and expressed in specific tissues and cells, making them particularly important as potential drug targets as >50% of therapeutic agents in the market target these receptors^19^.

To assess expression of the *OR52B4* gene in human eye tissue, we used online resource: “The Ocular Tissue Database”^20^. Expression of the *OR52B4* gene was present in the retina, optic nerve and choroid (Supplementary Table S4). The identification of olfactory gene expression in the eye is relatively new, but not surprising, as^21^ recently published expression profiling of a number of olfactory receptors (olfr) in mouse cornea, retina and choroid. Their analysis of eye tissue expression confirmed the presence of many olfr, with one of them, *olfr547* representing a homolog of the human *OR52B4* gene (85% identity). Interestingly, olfr547 is a close relative of olfr78, which has recently been shown to function in regulation of blood pressure in smooth muscle cells^22^,^23^. Expression of genes in the olfr pathway in mouse ocular tissues is mediated by the olfaction specific Gα protein, localised in the nuclear layer of the retina and previously thought to be only expressed in the olfactory epithelium^21^. Moreover, olfactory genes have been shown to be associated with cancer and other blood diseases, suggesting expression and function of olfactory proteins in a number of different pathways^23^,^24^,^25^. Interestingly, another olfactory receptor (*OR10J5*) was recently reported by *Kim and colleagues*^26^ to control cell migration and phosphorylation of signaling during angiogenesis in mice. However the functional role of *OR52B4* gene in angiogenesis needs to be elucidated and it has so far not previously been reported to be associated with pharmacogenetic response to AMD.

Previous studies investigating genetic factors in anti-VEGF treatment outcome have assessed known AMD risk-associated genes and genes encoding components of the neovascularization pathway^8^,^10^,^11^,^12^. However, most of these studies have produced variable results; for example, the Y402H variant in the *CFH* gene has been associated with poor VA outcome in some studies but in other studies has been reported to show no association with VA. Similarly, inconsistent results of association were reported for *ARMS2/HTRA1* 11,27. Our pooled GWAS findings did not identify genome-wide significance for SNPs in the previously associated known AMD risk genes *CFH, VEGF-A, HTRA1/ARMS2* (Supplementary Table S5). These findings are in agreement with our previous study^28^ and the CATT study, the largest current cohort in which major AMD risk alleles have been investigated for treatment outcome following treatment with bevacizumab, where no association with the *CFH, VEGF, VEGFR2* and *HTRA1*/*ARMS2* genes was identified with anti-VEGF treatment response^11^,^12^. To date, only one prior GWAS has been undertaken to identify genetic risk factors for treatment response to AMD. That study was conducted on 65 patients and reported the SNP rs7607942 near the *ERBB4* gene to be associated with poor VA outcome after 6 months of treatment, although this SNP did not reach genome-wide significance (uncorrected p = 6.692 × 10^−6^)^29^. Major drawbacks of that study were the small sample size and lack of replication of the reported association in an independent ranibizumab-treated AMD cohort. We did not find any association of this SNP in the current study (p > 0.05).

We chose to use a pooled DNA GWAS strategy to investigate response to anti-VEGF treatment in AMD as this provided a cost-effective and efficient approach to identify genetic associations^23^,^30^,^31^,^32^,^33^. We were able to technically validate ~70% of the 44 SNPs that were identified in the pooling strategy at a nominal significance level. Whilst most SNPs replicated, the level of significance of the SNPs diminished following technical validation. Possible explanations for the diminished significance may be due to experimental error during pool construction, array-specific errors resulting from the use of a limited number of arrays per pool, variation in allele frequency due to small sample size in some of the pools, allele-specific bias skewing the results for some SNPs, and/or small differences in the set of samples used in the validation stage^34^,^35^.

For the replication cohort, the number of injections at 6 month of treatment was not available, however it is interesting to note that a variation in treatment regime through change in number of injections between 3 and 6 months does not appear to ameliorate the genetic effect seen in the current study. However, after individual genotyping, replication and meta-analysis, SNP rs4910623 remained associated with worse functional outcome (change in VA) after the anti-VEGF treatment in AMD. Currently there is much interest in the role of anatomical features such as fluid clearance as a measure of treatment response. However, it was not considered in this analysis but this would be useful to examine in the future as to whether this SNP also affects fluid clearance and central macular thickness (CMT) on OCT following anti-VEGF treatment. To our knowledge, SNP rs4910623 and the 2 other SNPs associated with good response in the discovery phase have not previously been reported as associated with anti-VEGF treatment outcome in any type of cancer, thus it will be interesting to investigate the role of these SNPs in cancer following anti-VEGF treatment. Furthermore, much work needs to be undertaken to examine the biological basis of this phenomenon as well as the functional role of *OR52B4* gene in models of angiogenesis, such studies could lead to the development of other therapies for AMD patients that can be used as an adjunct to existing treatment. This, in turn, offers the prospect of personalising treatment, based on genotype and opens up the route for exploring other drug treatment opportunities. In conclusion, we report for the first time a pooled DNA based GWAS on pharmacogenetic response to ranibizumab treatment in AMD that identifies association of variant rs4910623 in the *OR52B4* gene. This finding was replicated in an independent replication cohort, suggesting that this gene may be involved in the response to anti-VEGF treatments in AMD patients of European decent. Finally, our data suggests that the SNP rs4910623 could be used as a diagnostic genetic marker before anti VEGF treatments for AMD.

## Methods

### Patient Recruitment and Data Collection

#### Melbourne Discovery Cohort

The study was approved by the Human Research and Ethics Committee of the Royal Victorian Eye and Ear Hospital (RVEEH) and performed in accordance to the tenets of the Declaration of Helsinki (7^th^ revision). Written informed consent was obtained for all study participants before participation. Patients were included in the study when they were >50 years of age, presented with an active sub-foveal CNV secondary to AMD, and received intravitreal anti-VEGF injections. Neovascularization was confirmed by fundus photography (Canon CR6-45NM; Canon Saitama, Japan), fundus fluorescein angiography (FFA) using IMAGEnet 2000 (Topcon Corporation, Tokyo, Japan), and optical coherence tomography (OCT) with Stratus OCT version 5.0.1 (Carl Zeiss Meditec, Dublin, CA) or Cirrus HD-OCT version 6.0.0.599 (Carl Zeiss Meditec). Exclusion criteria were nAMD associated with non-AMD conditions such as degenerative myopia, angioid streaks and hereditary retinal disorders. In addition bilateral nAMD patients who had different VA response between both eyes and treatment with either laser photocoagulation or photodynamic therapy before anti-VEGF treatment as previously reported were also excluded^36^. Initially a total of 315 nAMD patients were retrospectively recruited from the medical retinal clinic of the RVEEH. A total of 297 patients meet the eligibility criteria consisting of 277 patients with unilateral nAMD and 20 patients with bilateral nAMD who had the same (either gain or loss) VA response in both eyes. A total of 18 bilateral patients did not meet the eligibility criteria because they had differing VA response in both eyes.

VA was measured using an Early Treatment Diabetic Retinopathy Study (ETDRS) chart at 4-metre distance at each visit. The majority of nAMD patients were treated with monthly ranibizumab injections (Lucentis; Novartis Pharma AG, Basel, Switzerland) for 3 months of initial dosing except for 27 (9.0%) patients who first received a single injection of bevacizumab (Avastin; Roche, Basel, Switzerland) who then went on to receive normal dosing using ranibizumab. After 3 monthly injections, pro re nata treatment regimen were adopted for follow-up injections, that was based on evaluation by a retinal specialist, who considered loss of VA ≥ 5 EDTRS letters, the presence of fluid on an OCT scan or presence of chronic or new retinal haemorrhage as criteria for treatment. Data on demographics and clinical history (including VA and treatment regimens) were collected for all participating patients.

#### Replication Cohort

Ethical approval for the replication cohort was obtained from the local ethic committees of the Radboud university medical center, University of Cologne, and McGill University Health Centre. Written informed consent was acquired from all participants.

The replication cohort consisted of 376 treatment naïve patients of European descent with choroidal neovascularization secondary to AMD. Patients were diagnosed by retinal specialists based on ophthalmic examination, spectral-domain OCT (Spectralis HRA + OCT, Heidelberg Engineering, Heidelberg, Germany) or fluorescein angiography (FA) (Spectralis HRA + OCT, Heidelberg Engineering, Heidelberg, Germany; or Imagenet, Topcon Corporation, Tokyo, Japan). All included patients were over the age of 50 years, had not undergone any previous ophthalmic surgery, except for cataract removal, and did not have other retinal disorders besides AMD.

A total of 144 patients were treated at the Department of Ophthalmology of the Radboud university medical centre, Nijmegen, the Netherlands and 182 at the University of Cologne, Germany. These patients were included in the European Genetic Database (EUGENDA), a multicenter database for the clinical and molecular analysis of AMD. The remaining 50 patients were treated at the McGill University Health Center, Montreal, Canada. All patients received the loading dose of three consecutive monthly intravitreal injections of 0.5 mg ranibizumab (Lucentis; Novartis Pharma AG, Basel, Switzerland). Afterwards, they were followed up monthly and treated on a *pro re nata* regimen at the clinics of Nijmegen and Cologne and on a treat-and-extend regimen at the clinic of Montreal. In Nijmegen and Cologne (*pro re nata* regimen), in cases of persistence or recurrence of choroidal neovascularization, which was defined as loss of VA of ≥5 ETDRS letters, leakage seen on FA, fluid seen by OCT, or new macular hemorrhage or fluid, three consecutive monthly ranibizumab injections were administered. VA was assessed before treatment and after the three loading injections for all patients. VA was also recorded after 6 months of treatment for 262 patients. For 303 patients, Snellen VA measurements were recorded retrospectively and 73 patients were followed up prospectively using ETDRS VA. Baseline variables were collected using questionnaires or retrieved from the patient files. One eye was selected per patient; if both eyes received treatment, the first eye treated was selected and if treatment started simultaneously, the study eye was chosen randomly.

### First phase - Pooled DNA Based GWAS in the Melbourne Discovery Cohort

In the first phase of the study, a GWAS using a DNA pooling approach was conducted (Macgregor *et al*.^37^). A peripheral blood sample was collected from each patient and DNA was extracted (Qiagen, Victoria, Australia). Genomic DNA was quantified by Nanodrop Specrophotometer (Thermo Scientific) for a DNA concentration ≥175 ng/μl. A total of 285 samples were selected for the pooling cohort (12 samples were excluded due to a low DNA concentration required for pooling). Each pool was given a unique ID number, based on presenting VA. Non-responders from each pool were classified as those who showed loss of ≥5 EDTRS letters VA following 6 months of ranibizumab treatment and all remaining patients were classified as responders. Six equimolar DNA pools were generated with a pool size varying from 9 to 133 samples (Supplementary Table S2). An equal amount of DNA from each sample (varying from 0.2 μg to 0.6 μg) was added to the appropriate pool, with a minimum volume of 5 μl per sample to reduce pipetting errors at low volume. Pooled genomic DNAs were assayed using an Illumina Human Omni5-Quad Bead Chip that captured 4.3 million SNP markers. We randomised the pooled samples on the genotyping arrays to avoid spurious associations that may arise from array position. Approximately 4,600 indels or copy number variants were removed from the analyses. We applied stringent quality control (QC) as previously described (Lu *et al*.^38^). Briefly, we excluded SNPs with minor allele frequency (MAF) <1% from the reference panel of CEU (Utah residents with ancestry from northern and western Europe) samples in the 1000 Genome Project. Thus, the number of SNPs was reduced to 2.5 million. Further QC criteria for excluding SNPs from the analysis were: (1) SNPs with more than 10% negative probe scores; (2) a sum of the mean raw red and green intensity values less than 1200 for each array (to ensure calibration); (3) SNPs with a small number of expected probes in pools (SNPs with MAF between 1–5% required at least half of the expected number of probes, and SNPs with MAF > 5% required at least a third; the expected number of probes was calculated as the average number of probes per pool per SNP multiplied by the number of samples in each pool); (4) non-autosomal SNPs; (5) SNPs where the variance of the estimated allele frequency was significantly different between all pools. After applying these QC filters, a total of 1,940,408 SNPs were retained for further analysis. A GWAS was carried out between responders and non-responders from three different baseline VA categories and then results were combined in a meta-analysis, which included 225 responders and 60 non-responders.

### Second Phase - Technical Replication in the Melbourne Discovery Cohort

In the 2^nd^ phase, the most interesting associations found in the pooled DNA GWAS were validated by undertaking individual genotyping of the original 285 samples, plus the inclusion of the additional 12 patient samples that subsequently became available whose initial concentration was not enough for the pooling GWAS. These samples were genotyped individually for 44 SNPs using the MassArray platform (Agena Bioscience, San Diego, CA) and performed as previously described^36^.

### Third Phase - Replication in an Independent Cohort of European Descent

In the third phase of the study, SNPs that showed association (p < 0.05 after correction for multiple testing) in the 2^nd^ phase of the study were analysed in an independent ranibizumab-treated AMD cohort of European descent. DNA was extracted and quantified in Nijmegen, the Netherlands. Subsequently, DNA samples of a total of 376 AMD patients were genotyped in Melbourne using the MassArray platform (Agena Bioscience, San Diego, CA) as previously described^36^.

### Statistical analysis

Linear regression was undertaken to assess the role of non-genetic factors (potential cofounders), including age, sex, smoking status, CNV size, lesion type, number of injections and baseline VA, on the dependent variable “change in VA” at 3 and 6 months respectively.

The GWAS of pooled DNA was undertaken at the Queensland Institute of Medical Research (QIMR) as previously described^37^. A customised pipeline developed at QIMR was used for data analysis^37^,^38^. Briefly, a GWAS was run within each pool comparison, i.e. a linear model based approach was used for association between SNP and allele frequency difference in responder compared to non-responder pools accounting for pooling errors. A meta-analysis was performed summarising the GWAS results from the multiple pool comparisons, weighted by inverse variances. Finally, we applied post-analysis checks to the meta-analysis results in order to reduce the chance of false positive findings, filtering any SNP that had results from only one of the multiple pool comparisons and those with large discrepancies in association results of the SNP itself and its proxies.

For individual genotyping data, logistic regression was used to assess differences in allele frequency between the responder and non-responder groups (defined in the same manner as for the GWAS) and adjusted for baseline VA in the discovery cohort and baseline VA and age at first injection in the replication cohort. In this replication cohort, different treatment regimens were administered after the 3 initial monthly injections. Therefore, we stratified the analysis for the replication cohort into two groups; pro-re-nata and treat-and-extend. These groups consisted of 215 pro re nata (Nijmegen and Cologne) and 47 treat-and-extend (Montreal) patients respectively. Results are reported as odds ratios (OR), 95% confidence intervals (95% CI) and p value with statistical significance being defined as *p* < 0.05 following Bonferroni correction for multiple testing. For the analyses, the statistical software Plink V1.07 (http://pngu.mgh.harvard.edu/~purcell/plink/) and SPSS IBM SPSS Statistics for Windows, version 20.0 (IBM Corp., Armonk, New York, USA) were used. To prevent any possible confounding, the analyses performed in the both the Melbourne discovery and the replication cohort were corrected for baseline VA.

Meta-analysis of the discovery and replication cohorts was performed using Metal software^39^. For 3 month meta-analysis all the patients from Melbourne discovery and the replication cohort were included because they all were treated with 3 initial doses of anti-VEGF injections while for the 6 months analysis we considered the fact that the Melbourne discovery cohort was treated using a pro re nata strategy. Therefore, we included only the 215 pro re nata treated patients from Nijmegen and Cologne and excluded the 47 patients from Montreal who were treated with a treat-and-extend regimen.

## Additional Information

**How to cite this article**: Riaz, M. *et al*. GWAS study using DNA pooling strategy identifies association of variant rs4910623 in *OR52B4* gene with anti-VEGF treatment response in age-related macular degeneration. *Sci. Rep.* **6**, 37924; doi: 10.1038/srep37924 (2016).

**Publisher's note:** Springer Nature remains neutral with regard to jurisdictional claims in published maps and institutional affiliations.

## Supplementary Material

Supplementary Information

## Figures and Tables

**Table 1:** Patient demographics and clinical characteristics of the Melbourne discovery cohort and the replication cohorts.

	Discovery Cohort (Melbourne) (n = 297)	Replication Cohort (Nijmegen/Cologne/Montreal) (n = 376)
Sex		
Female	181 (60.9%)	214 (56.9%)
Male	116 (39.1%)	162 (43.1%)
Age (years), mean ± SD Range	79.2 ± 7.1 (53–102)	77.1 ± 7.4 (53–97)
Baseline Visual Acuity (ETDRS letters), mean ± SD Range	51.0 ± 17.5 (2–88)	52.6 ± 18.0^∞^ (1–85)
Type of lesion		
Predominantly Classic	23%	19.4%
Non-Predominantly Classic	77%	69.7%
Missing Data	11%	10.9%
*^+^Size of CNV		
<2 optic-disc area	44%	24.5%
>2 Optic-disc area	29%	51.3%
Missing Data	27%	24.2%
Number of Injections at 6 months, mean ± SD	4.7 ± 1.3	NA
^Number of Responders/Non-responders		
3 Month	84%/16%	82%/18%
6 Month	79%/21%	76%/24%

SD = Standard Deviation, ETDRS = Early Treatment Diabetic Retinopathy Study, *In the discovery cohort, one optic-disc area is equal to 2.54 mm^2^, based on an optic disc diameter of 1.8 mm, ^+^In the replication cohort optic disc measurement is based on each patient optic disc area, NA = Not available, ^Non-responders classified as those who showed loss of ≥5 EDTRS letters VA from baseline; all remaining patients were classified as responders. ^∞^For 303 patients, Snellen visual values were first converted into approximate ETDRS letters using a chart that has all three measurement to read the equivalent number of ETDRS letters. It is based on formula ETDRS letters = 85 − ((−(logSnellen))/0.02)^40^.

**Table 2:** Selected SNPs from the pooled GWAS comparing responders and non-responders at 6 months ranibuzimab treatment and validation results using an independent genotyping technique in the Melbourne discovery cohort.

SNP	Chr.	Position^+^	Gene	Effect Allele	Pooled GWAS		Technical Replication	
Pooled GWAS SNPs reaching P ≤ 9 × 10^−8^					*P Value*	OR	*P Value*	OR (95% CI)
**rs4910623**	**11**	**4389639**	***OR52B4***	**G**	**3.69 × 10**^**−9**^	**3.14**	**5.65 × 10**^**−5**^	**2.58 (1.63–4.10)**
**rs323085**	**18**	**49290621**	***LOC100287225***	**C**	**3.29 × 10**^**−8**^	**0.22**	**6.53 × 10**^**−4**^	**0.16 (0.06–0.46)**
**rs10158937**	**1**	**66144876**	***LEPR***	**C**	**5.36 × 10**^**−8**^	**0.28**	**1.30 × 10**^**−3**^	**0.32 (0.17–0.65)**
rs2475779	5	157541895	*CLINT1*	T	1.59 × 10^−9^	0.29	1.36 × 10^−3^	0.40 (0.23–0.70)
rs59741976	3	74593662	*CNTN3*	T	2.65 × 10^−8^	0.32	1.84 × 10^−3^	0.31 (0.15–0.65)
rs4655583	1	66155407	*LEPR*	A	1.37 × 10^−8^	0.28	2.38 × 10^−3^	0.34 (0.17–0.69)
rs7857431	9	132529504	*PTGES*	T	3.36 × 10^−8^	0.25	4.58 × 10^−3^	0.12 (0.03–0.53)
rs794009	4	40139719	*N4BP2*	A	6.91 × 10^−8^	0.35	5.47 × 10^−3^	0.40 (0.22–0.77)
rs10234065	7	19547138	*TWISTNB*	T	1.37 × 10^−8^	0.31	5.61 × 10^−3^	0.13 (0.03–0.56)
rs1447830	3	74613171	*CNTN3*	T	7.56 × 10^−9^	0.30	5.63 × 10^−3^	0.35 (0.17–0.74)
rs2110470	7	19509870	*TWISTNB*	A	3.35 × 10^−8^	0.35	5.65 × 10^−3^	0.40 (0.21–0.77)
rs1892535	1	66097181	*LEPR*	T	6.50 × 10^−8^	0.31	6.50 × 10^−3^	0.38 (0.19–0.77)
rs1573317	18	382268	*COLEC12*	T	1.11 × 10^−9^	0.27	7.60 × 10^−3^	0.36 (0.18–0.77)
rs13154178	5	42828101	*SEPP1*	A	2.09 × 10^−8^	0.36	8.20 × 10^−3^	0.49 (0.30–0.83)
rs4909963	11	11119228	*GALNTL4*	T	9.85 × 10^−9^	0.24	8.32 × 10^−3^	0.20 (0.06–0.67)
rs79966776	3	74582701	*CNTN3*	A	1.61 × 10^−8^	0.32	9.72 × 10^−3^	0.42 (0.22–0.81)
rs510549	3	111700305	*ABHD10*	T	1.60 × 10^−9^	0.34	1.07 × 10^−2^	0.53 (0.33–0.87)
rs6917419	6	27243480	*PRSS16*	T	3.80 × 10^−8^	0.33	1.41 × 10^−2^	0.32 (0.13–0.80)
rs12117294	1	209814879	*LAMB3*	T	1.89 × 10^−8^	0.34	1.84 × 10^−2^	0.46 (0.25–0.88)
rs10767060	11	23468443	*LOC100500938*	T	8.72 × 10^−8^	0.38	2.23 × 10^−2^	0.58 (0.37–093)
rs17770298	9	101208288	*GABBR2*	A	7.44 × 10^−9^	0.18	2.40 × 10^−2^	0.19 (0.05–081)
rs11131078	3	7548067	*GRM7*	T	3.23 × 10^−9^	0.13	2.63 × 10^−2^	0.58 (0.37–0.94)
rs292998	5	58032485	*RAB3C*	G	3.86 × 10^−9^	0.38	2.94 × 10^−2^	0.61 (0.39–0.95)
rs772433	2	7838257	*LOC339788*	A	5.06 × 10^−8^	0.36	4.24 × 10^−2^	0.62 (0.40–0.98)
rs3806586	2	128433897	*LIMS2*	T	1.16 × 10^−9^	0.26	4.96 × 10^−2^	0.52 (0.28–1.00)
rs9644866	9	2290590	*SMARCA2*	T	3.72 × 10^−9^	0.17	5.60 × 10^−2^	0.63 (0.40–1.01)
rs10050214	4	78733015	*CNOT6L*	T	4.22 × 10^−8^	0.38	6.35 × 10^−2^	0.40 (0.28–1.04)
rs12638297	3	29748169	*RBMS3*	T	4.29 × 10^−8^	0.31	6.40 × 10^−2^	0.50 (0.25–1.04)
rs4449299	3	14598965	*GRIP2*	A	5.50 × 10^−8^	0.39	8.79 × 10^−2^	0.64 (0.39–1.07)
rs1353892	12	90716019	*C12orf37*	T	1.06 × 10^−9^	0.34	9.71 × 10^−2^	0.67 (0.42–1.07)
rs659910	11	131769454	*NTM*	T	1.27 × 10^−8^	0.39	1.67 × 10^−1^	0.71 (1.00–1.03)
rs10141328	14	96752555	*ATG2B*	C	7.78 × 10^−8^	0.16	1.90 × 10^−1^	0.73 (0.47–1.16)
rs9323992	14	98649816	*C14orf64*	T	4.59 × 10^−8^	0.38	5.14 × 10^−1^	0.84 (0.50–1.41)
rs7432690	3	77450363	*ROBO2*	T	9.91 × 10^−10^	0.14	6.81 × 10^−1^	1.09 (0.72–1.67)
rs141659302	11	128802177	*TP53AIP1*	T	3.79 × 10^−8^	0.41	9.99 × 10^−1^	0.74 (0.00–0.00)
rs7320683	13	71787948	*DACH1*	A	5.38 × 10^−8^	2.86	1.00	1 (−)
rs291477	3	73807858	*PDZRN3*	G	1.85 × 10^−10^	2.91	1.00	1 (−)
**Pooled GWAS SNPs in known pharmagenes showing suggestive significance**								
rs3804938	3	7550294	*GRM7*	C	1.35 × 10^−7^	0.13	1.01 × 10^−2^	0.54 (0.34–0.87)
rs4148732	7	87234049	*ABCB1*	G	2.72 × 10^−6^	0.22	1.00 × 10^−3^	0.08 (0.02–0.38)
**Pooled GWAS SNPs leading to a missense change in gene**								
rs3877899	5	42801268	*SEPP1*	A	9.02 × 10^−7^	0.36	1.15 × 10^−3^	0.34 (0.18–0.66)
rs34677	5	33998768	*AMACR*	T	3.22 × 10^−5^	0.35	3.0 × 10^−3^	0.26 (0.11–064)
rs3784588	15	31294654	*TRPM1*	T	4.73 × 10^−5^	0.27	9.96 × 10^−1^	0.04 (1.00–1.03)
rs17659179	18	47511113	*MYO5B*	A	8.20 × 10^−5^	0.28	4.75 × 10^−2^	0.29 (0.09–0.99)
rs17673268	9	368128	*DOCK8*	T	1.54 × 10^−5^	0.41	2.44 × 10^−1^	0.63 (1.00–1.03)

For technical replication P values were calculated using logistic regression adjusted for baseline visual acuity, OR = odds ratio, SNP: single nucleotide polymorphism, Chr: chromosome, BP: base pair position on chromosome. SNPs that remained significantly associated after Bonferroni correction for multiple testing are highlighted in bold. ^+^Representing position from phase 3 1000 G CEU reference panel.

**Table 3:** Genotypic distribution and allele frequency of rs4910623, rs323085 and rs10158937 and association with VA response at six and three months of anti-VEGF treatment in the Melbourne discovery cohort.

**6 Month**								
SNP	A1	A2	Genotype frequency*		Allele frequency ^+^		P-Value	OR 95% CI
			Responders	Non-responders	Responders	Non-Responders		
rs4910623	**G**	A	0.23/0.52/0.25	0.54/0.36/0.10	0.49/0.51	0.72/0.28	**5.7 × 10**^**−5**^	2.58 (1.63–4.10)
rs323085	**C**	T	0.30/0.28/0.69	0/0.70/0.93	0.17/0.83	0.03/0.97	6.5 × 10^**−**4^	0.16 (0.06–0.46)
rs10158937	**C**	A	0.06/0.33/0.61	0/0.17/0.83	0.23/0.77	0.08/0.92	1.3 × 10^**−**3^	0.32 (0.17–0.65)
**3 Month**								
rs4910623	**G**	A	0.25/0.51/0.24	0.52/0.37/0.11	0.51/0.49	0.71/0.29	**1.5 × 10**^**−3**^	2.23 (1.36–3.66)
rs323085	**C**	T	0.02/0.25/0.73	0.02/0.22/0.76	0.14/0.86	0.13/0.87	0.81	0.92 (0.47–1.81)
rs10158937	**C**	A	0.06/0.33/0.62	0.02/0.13/0.85	0.22/0.78	0.09/0.91	9.0 × 10^**−**3^	0.36 (0.17–0.77)

Definition of response was the same as in the pooled GWAS: Non-responders are patients who lost ≥5 ETDRS letters and the remainder were classified as responders P-Value: Calculated using logistic regression adjusted for baseline visual acuity, OR = odd ratio, representing trend per copy effect of risk or minor allele on VA outcome, highlighted in bold for each SNP, CI: Confidence Interval, ^*^Genotype frequency A1A1/A1A2/A2A2, ^**+**^Allele frequency A1/A2.

**Table 4:** Genotypic distribution and allele frequency of rs4910623, rs323085 and rs10158937 and association with VA response at six and three months of anti-VEGF treatment in the replication cohort.

6 Month^++^								
SNP	A1	A2	Genotype frequency*		Allele frequency^+^		P-Value	OR 95% CI
			Responders	Non-responders	Responders	Non-Responders		
rs4910623	**G**	A	0.24/0.48/0.28	0.34/0.54/0.12	0.48/0.52	0.62/0.38	**3.5 × 10**^**−2**^	1.71 (1.04–2.80)
rs323085	**C**	T	0.02/0.25/0.74	0.00/0.21/0.79	0.14/0.86	0.10/0.90	0.46	0.74 (0.34–1.63)
rs10158937	**C**	A	0.05/0.32/0.63	0.02/0.30/0.67	0.21/0.79	0.17/0.83	0.58	0.84 (0.45–1.57)
**3 Month**								
rs4910623	**G**	A	0.22/0.50/0.28	0.39/0.46/0.15	0.47/0.53	0.62/0.38	**2.4 × 10**^**−3**^	1.83 (1.24–2.71)
rs323085	**C**	T	0.03/0.24/0.73	0/0.23/0.77	0.15/0.85	0.11/0.89	0.34	0.74 (0.50–1.33)
rs10158937	**C**	A	0.04/0.32/0.64	0.06/0.35/0.59	0.20/0.80	0.23/0.77	0.25	1.31 (0.83–2.06)

Definition of response was the same as in the pooled GWAS: Non-responders are patients who have lost ≥5 ETDRS letters and the remainder were classified as responders P-Value: Calculated using logistic regression adjusted for baseline visual acuity and age, OR = odd ratio, representing trend per copy effect of risk or minor allele on VA outcome, highlighted in bold for each SNP, CI: Confidence Interval, ^*^Genotype frequency A1A1/A1A2/A2A2, ^+^Allele frequency A1/A2. ^++^In replication cohort pro re nata treated patients included n = 215.

**Table 5:** Meta-analysis of the Melbourne discovery cohort and the replication cohort for SNP rs4910623 at 3 and 6 months of ranibizumab treatment in AMD patients.

Meta-Analysis											
SNP	Gene	A1	A2	3 Month				6 Month*			
				P value	N	Direction	Z score	P value	N	Direction	Z score
rs4910623	*OR52B4*	G	A	1.2 × 10^−5^	673	++	4.37	9.3 × 10^−6^	512	++	4.43

Meta-analysis was performed using METAL. ^*^AT 6 month only pro re nata treated patient were included from replication cohort.
