# Supplementary material for: GWAS study using DNA pooling strategy identifies association of variant rs4910623 in OR52B4 gene with anti-VEGF treatment response in age-related macular degeneration
Source: Sci Rep. 2016 Nov 28;6:37924. doi: 10.1038/srep37924 (PMC5124940; doi:10.1038/srep37924)
Supplement: Supplementary Information [file srep37924-s1.pdf]

GWAS using DNA pooling identifies association of rs4910623 in the *OR52B4* gene with anti-VEGF treatment response in age-related macular degeneration

Moeen Riaz[1,2£], Laura Lorés-Motta[3£], Andrea J. Richardson[1,2], Yi Lu[4], Grant Montgomery[5], Amer Omar[6], Robert K. Koenekoop[7], John Chen[8], Philipp Muether[9], Lebriz Altay[9], Tina Schick[9], Sascha Fauser[9], Dzenita Smailhodzic[3], Freekje van Asten[3], Eiko K. de Jong[3], Carel B. Hoyng[3], Kathryn P Burdon[10], Stuart MacGregor[4], Robyn H. Guymer[1,2], Anneke I. den Hollander[3,11], Paul N Baird[*1,2]

1. Centre for Eye Research Australia, Royal Victorian Eye and Ear Hospital.
2. Ophthalmology, Department of Surgery, University of Melbourne.
3. Department of Ophthalmology, Donders Institute for Brain, Cognition and Behaviour, Radboud university medical center, Nijmegen, the Netherlands
4. Statistical Genetics Laboratory, QIMR Berghofer Medical Research Institute, Brisbane, Australia.
5. Molecular Epidemiology, QIMR Berghofer Medical Research Institute, Brisbane, Australia.
6. Montreal Retina Institute, Westmount Canada.
7. Paediatric Surgery, Human Genetics, and Ophthalmology, McGill University Health Centre, Montreal, Quebec, Canada.
8. Department of Ophthalmology, McGill University Health Centre, Montreal, Quebec, Canada.
9. Department of Ophthalmology, University Hospital of Cologne, Cologne, Germany
10. Menzies Institute for Medical Research, University of Tasmania, Hobart, TAS and Dept: Ophthalmology, Flinders University, Adelaide, SA
11. Department of Human Genetics, Radboud university medical center, Nijmegen, the Netherlands.

[£]These authors contributed equally to this work.

Correspondence to: Paul N. Baird [pnb@unimelb.edu.au]

Table S1: Analysis of the influence of demographic and clinical variables on the change in VA after 3 and 6 month of anti-VEGF treatment in the Melbourne discovery cohort and the replication Cohort

	Melbourne Discovery Cohort				Replication Cohort			
	3 Months		6 Months		3 Months		6 Months	
**Characteristics**	**P-Value**	**β (CI 95%) SE**	**P-Value**	**β (CI 95%) SE**	**P-Value**	**β (CI 95%) SE**	**P-Value**	**β (CI 95%) SE**
Gender (Male vs Female)	0.86	-0.26(-2.7-2.1)0.93	0.84	-0.311(-3.4-2.84)1.58	0.89	0.33 (-1.13 - 4.78) 2.26	0.33	2.78 (-2.88 – 8.45) 2.86
Age	0.27	-1.10(-0.2-0.05)0.10	0.45	-0.07(-0.2-0.13)0.10	**<0.01**	-0.42 (-0.70 - -0.13) 0.14	0.06	-0.37 (-0.75 – 0.11) 0.19
Baseline VA	**<0.001**	-0.24(-0.3 to -0.17)0.03	**<0.001**	-0.32 (-0.32 to -0.15)0.04	**0.001**	-0.21 (-0.33 - -0.09) 0.06	**0.04**	-0.18 (-0.35 – -0.007) 0.09
Smoking	0.71	0.53(-1.9-2.9)1.48	0.42	1.27(-1.84 4.38) 1.58	0.33	2.21 (-2.29 - 6.71) 2.28	0.37	2.61 (-3.14 – 8.36) 2.90
Type of lesion	0.30	2.1(-1.2-5.5)2.0	0.45	-1.74(-6.2 to 2.78)2.2	0.63	1.23 (-3.87 – 6.33) 2.58	0.50	-2.21 (-8.68 – 4.27) 3.27
Size of CNV	0.91	0.18(-2.8-3.2)1.83	0.53	-1.27(-5.2 to 2.73)2.0	0.12	-0.48 (-1.08 – 0.133) 0.31	0.08	-0.71 (-1.51 - 0.09) 0.41
Number of injections	-	-	0.25	-3.4(-5.9 to -1.9)0.71	-	-	-	-

P value calculated using a linear regression test for change in VA and non-genetic covariates, β: coefficient of the linear regression, CI: confidence Interval, SE: Standard Error, VA: Visual Acuity, CNV: Choroidal Neovascularization.

Table S2: Design of DNA pools, pool comparisons and meta-analysis in pooled GWAS in the Melbourne discovery cohort (1st phase of study).

Pool ID (responder)	Presenting VA ETDRS Letters	Pool size	Male/Female n (%)	Mean age ± SD (years)		Pool ID (non-responders)	Presenting VA ETDRS Letters	Pool size	Male/Female N (%)	Mean age ± SD (years)
1	= 36 -69	133	48(17.0)/85(29.9)	78.9±6.3		2	=36 -69	44	21(7.4)/23(8.0)	79.0±8.6
3	≤ 35	46	23(8.0)/23(8.0)	79.9±7.0	**VS**	4	≤ 35	7	1(0.3)/6(2.1)	80.8±7.6
5	≥ 70	46	19(6.6)/27(9.5)	79.8±7.7		6	≥ 70	9	3(1.1)/6(2.1)	80.7±8.0

**Meta-analysis of pool comparisons**	**Responder/Non-responders**
1, 3,5 vs 2,4,6	(225 individuals)/ (60 individuals)

Different pool sizes lead to differences in pooling variance; thus pools were combined to have similar sizes (responders checked for association against non-responders). Mean age (at time of first treatment with anti-VEGF). SD=standard deviation

Table S3: HaploReg database showing variants located within predicted regulatory regions and in LD ($r^2 > 0.8$) with rs4910623 (+/- 250Kb)

Chr	pos (hg19)	LD(r^2)	LD(D')	Variant	Ref	Alt	EUR (freq)	Motifs Changed	RefSeq Genes	Functional Annotation
11	4372783	0.87	0.96	rs4910613	A	C	0.49	-	16kb 3' of OR52B4	-
11	4377981	0.94	0.98	rs10835954	C	T	0.5	Pou5f1	11kb 3' of OR52B4	-
11	4387760	0.99	1	rs12293167	G	T	0.5	5 altered motifs	731bp 3' of OR52B4	-
11	4389587	1	1	rs7929171	G	C	0.51	DMRT1,DMRT4,LUN-1	OR52B4	5'-UTR
**11**	**4389639**	**1**	**1**	**rs4910623**	**G**	**A**	**0.51**	**GATA,TCF4**	**22bp 5' of OR52B4**	-
11	4395551	0.91	-0.98	rs1895914	C	T	0.51	Nkx2,Sox	5.9kb 5' of OR52B4	-
11	4396029	0.92	-0.99	rs12575038	T	C	0.51	TR4	6.4kb 5' of OR52B4	-
11	4399936	0.88	-0.99	rs7124081	A	C	0.52	CEBPB	6.2kb 3' of TRIM21	

HaploReg http://www.broadinstitute.org/mammals/haploreg/haploreg.php

Table S4: The Ocular Tissue Database shows expression of the *OR52B4* gene in adult human eye tissues

Seq name	start	stop	strand	Probe Id	GENE	[1]PLEIR Score in different tissues of Eye			
						Choroid & RPE	Optic nerve	Optic nerve head	Retina
chr11	4388614	4391330	-	3360136	OR52B4	17.3781	22.4866	22.9475	25.5724

1: Expression of genes in the Ocular Tissue Database (https://genome.uiowa.edu/otdb/) is represented as an Affymetrix Probe Logarithmic Intensity Error (PLIER) number.RPE: retinal pigment epithelium

Table S5: Pooled GWAS association of previously associated known AMD risk genes with change in VA at 6 month of anti-VEGF treatment in Melbourne discovery cohort

GENE	SNP	P-value
VEGF-A		
	rs699946	0.24
	rs3025000	0.04
ARMS2		
	rs10490924	0.85
HTRA1		
	rs2284665+	0.09
	rs932275+	0.06
CFH		
	rs3753394	0.54
	rs800292	0.33
	rs1065489	0.23
	rs7529589*	0.55

+SNPs in high LD with rs11200638 (r^2>0.8)
*SNP in high LD with rs1061170 (r^2=1)
